# Readability of Wikipedia Pages on Autoimmune Disorders: Systematic Quantitative Assessment

**DOI:** 10.2196/jmir.8225

**Published:** 2017-07-18

**Authors:** Abdulla Watad, Nicola Luigi Bragazzi, Francesco Brigo, Kassem Sharif, Howard Amital, Dennis McGonagle, Yehuda Shoenfeld, Mohammad Adawi

**Affiliations:** ^1^ Department of Medicine B Sheba Medical Centre Tel Aviv Israel; ^2^ Sackler Faculty of Medicine Tel Aviv University Tel Aviv Israel; ^3^ Zabludowicz Center for Autoimmune Diseases Sheba Medical Centre Tel Aviv Israel; ^4^ Department of Health Sciences University of Genoa Genoa Italy; ^5^ Department of Neuroscience University of Verona Verona Italy; ^6^ Leeds Institute of Rheumatic and Musculoskeletal Medicine University of Leeds Leeds United Kingdom; ^7^ Bar-Ilan Faculty of Medicine Padeh and Ziv Hospitals Zefat Israel

**Keywords:** autoimmune diseases, eHealth, telemedicine, readability, Wikipedia

## Abstract

**Background:**

In the era of new information and communication technologies, the Internet is being increasingly accessed for health-related information. Indeed, recently published patient surveys of people with autoimmune disorders confirmed that the Internet was reported as one of the most important health information sources. Wikipedia, a free online encyclopedia launched in 2001, is generally one of the most visited websites worldwide and is often consulted for health-related information.

**Objective:**

The main objective of this investigation was to quantitatively assess whether the Wikipedia pages related to autoimmune disorders can be easily accessed by patients and their families, in terms of readability.

**Methods:**

We obtained and downloaded a list of autoimmune disorders from the American Autoimmune Related Diseases Association (AARDA) website. We analyzed Wikipedia articles for their overall level of readability with 6 different quantitative readability scales: (1) the Flesch Reading Ease, (2) the Gunning Fog Index, (3) the Coleman-Liau Index, (4) the Flesch-Kincaid Grade Level, (5) the Automated Readability Index (ARI), and (6) the Simple Measure of Gobbledygook (SMOG). Further, we investigated the correlation between readability and clinical, pathological, and epidemiological parameters. Moreover, each Wikipedia analysis was assessed according to its content, breaking down the readability indices by main topic of each part (namely, pathogenesis, treatment, diagnosis, and prognosis plus a section containing paragraphs not falling into any of the previous categories).

**Results:**

We retrieved 134 diseases from the AARDA website. The Flesch Reading Ease yielded a mean score of 24.34 (SD 10.73), indicating that the sites were very difficult to read and best understood by university graduates, while mean Gunning Fog Index and ARI scores were 16.87 (SD 2.03) and 14.06 (SD 2.12), respectively. The Coleman-Liau Index and the Flesch-Kincaid Grade Level yielded mean scores of 14.48 (SD 1.57) and 14.86 (1.95), respectively, while the mean SMOG score was 15.38 (SD 1.37). All the readability indices confirmed that the sites were suitable for a university graduate reading level. We found no correlation between readability and clinical, pathological, and epidemiological parameters. Differences among the different sections of the Wikipedia pages were statistically significant.

**Conclusions:**

Wikipedia pages related to autoimmune disorders are characterized by a low level of readability. The onus is, therefore, on physicians and health authorities to improve the health literacy skills of patients and their families and to create, together with patients themselves, disease-specific readable sites, disseminating highly accessible health-related online information, in terms of both clarity and conciseness.

## Introduction

In the era of so-called eHealth, characterized by the spreading of new information and communication technologies, including the dynamic Web 2.0, every day millions of people surf the Internet as a source of health information [1-4]. They search for online material concerning the symptoms and clinical manifestation of a recently diagnosed disease, its possible management, the adverse effects of treatments, the details of diagnostic procedures, and its prognosis.

Recent surveys conducted among patients with autoimmune disorders found the Internet to be one of the most important sources of health information on their condition [5]: YouTube, for example, is a source of information on rheumatoid arthritis, with a wide viewership and a potential to affect patients’ knowledge and attitudes [6]. Another study found that Web activities were influenced by the media coverage and publicity about a celebrity’s illness. In particular, the death of Harold Allen Ramis, a famous American actor, director, writer, and comedian, due to complications of an autoimmune inflammatory vasculitis, resulted in an increase in vasculitis-related Google searches, Wikipedia page accesses, and tweet production, peaking in February 2014 [7].

The need for high-quality, accurate, but at the same time freely and easily accessible health care information on autoimmune disorders to better inform patients, their families, and the general population is of crucial and urgent importance, in that these disorders are characterized by a relevant societal and clinical burden [8]. The European Commission has acknowledged readability, that is to say the legibility of a written text and the ease with which a reader can understand and comprehend it, and accessibility as one of the 6 quality criteria of health-related websites [9].

Since its launch in 2001, the free online encyclopedia Wikipedia has become the most popular general reference site on the Internet, and it is constantly accessed as a popular source of health care-related information. Wikipedia contains more than 30 million articles, which are available in up to 287 languages, including over 4.6 million English-language articles [10]. With the impressive figure of more than 18 billion page views and nearly 500 million unique visitors per month, the English version of Wikipedia ranks fifth in the list of most surfed websites worldwide [11].

Noteworthy, most Wikipedia articles dedicated to autoimmune disorders rank first or among the first results in a Google search. As such, these articles are highly likely to be one of the most read and consulted sources of online information on autoimmune disorders for millions of English-speaking Internet users globally. The aim of this study was to quantitatively assess the readability of Wikipedia pages related to autoimmune disorders, using a systematic search strategy and validated readability instruments.

## Methods

We obtained and downloaded a list of autoimmune disorders from the American Autoimmune Related Diseases Association (AARDA) website [12].

For each autoimmune disorder, we retrieved and downloaded the corresponding Wikipedia page for further processing.

In particular, we removed sections such as copyright information, references, and images from all pages. We analyzed Wikipedia articles for their overall level of readability using 6 different quantitative readability scales: (1) the Flesch Reading Ease, (2) the Gunning Fog Index, (3) the Coleman-Liau Index, (4) the Flesch-Kincaid Grade Level, (5) the Automated Readability Index (ARI), and (6) the Simple Measure of Gobbledygook (SMOG).

Generally speaking, readability scores reflect parameters like sentence length, number of sentences, and the number of syllables or characters per word. Generally, polysyllabic words, long, complex sentences, and articulated paragraphs are penalized.

In more detail, the Flesch Reading Ease readability index reports readability scores ranging from 0 to 100, with higher scores indicating a more easily accessible and readable text. Material with a score of 70 is usually considered to be appropriate for most adults, while text with a score between 30 and 50 is considered difficult to read, and text with a score between 50 and 60 is perceived as fairly difficult.

The other 5 readability indexes—the Gunning Fog Index, the Coleman-Liau Index, the Flesch-Kincaid Grade Level, the ARI, and the SMOG—report a number that corresponds to an academic grade level (ie, to the number of years of formal education that a person would need in order to be able to understand the text easily on the first reading). The Gunning Fog Index is based on average sentence length and the number of complex words: a value less than 12 indicates a text that can be read and understood by a wide audience, being universally understandable if the value is less than 8. The Coleman-Liau Index is based on the average numbers of letters and the average number of sentences: a text whose score is less than 7 can be universally comprehended. The Flesch-Kincaid Grade Level relies on the average number of words and the average number of syllables: a text with a score of 7-8 is legible by a wide audience. The ARI is based on the average number of letters and the average number of words: a score less than 14 indicates a text understandable by a wide audience, while a value of 14 indicates a text that requires a university education. The SMOG is based on the average number of polysyllables and the average number of sentences: a commonly recommended grade level is in the range 7-8.

Further, we analyzed eventual correlations between readability scores and epidemiological and clinical parameters of the autoimmune disorders under study. In particular, we investigated the following parameters: age at onset (≤20 years, 20-40 years, 40-60 years, 60-80 years); the incidence and prevalence figures according to the literature; clinical and pathological features (organ-specific vs systemic disease); and the McGonagle classification (classic polygenic autoimmune disease; polygenic autoinflammatory disease; mixed-pattern disease) [13]. We hypothesized that Wikipedia pages describing complex, unfamiliar, and less common diseases would be characterized by lower readability scores than would Wikipedia articles focusing on more common disorders. Further, since readability requires literacy skills, we expected a certain degree of correlation with onset age.

We computed continuous data as mean (SD). For investigating the correlation between readability and clinical, pathological, and epidemiological parameters, we performed analysis of variance or Student *t* test as univariate analyses. For multivariate analysis, we carried out regression analyses.

Further, we analyzed each Wikipedia page taking into account its different sections focusing on the following: pathogenesis of the disease (termed “pathogenesis”), management (termed “treatment”), diagnosis (termed “diagnosis”), and prognosis (termed “prognosis”). Paragraphs not falling into any of these categories were grouped in a section termed “other.”

We conducted statistical analyses using the commercial software IBM SPSS version 23.0 (IBM Corporation). Graphs were generated using the commercial software MedCalc Statistical Software version 16.8.4 (MedCalc Software bvba).

## Results

We retrieved 134 diseases from the AARDA website. Table 1 reports reading level assessments of the autoimmune disorder-related Wikipedia pages we analyzed. The mean Flesch Reading Ease score indicated that Wikipedia pages related to autoimmune disorders would require at least a university graduate school level, being very difficult to read and understand. The other readability scores suggested that overall readability corresponded to a 14th to 15th academic grade level. All the readability indices confirmed that the sites were suitable for a university graduate reading level (Table 1).

**Figure 1:**
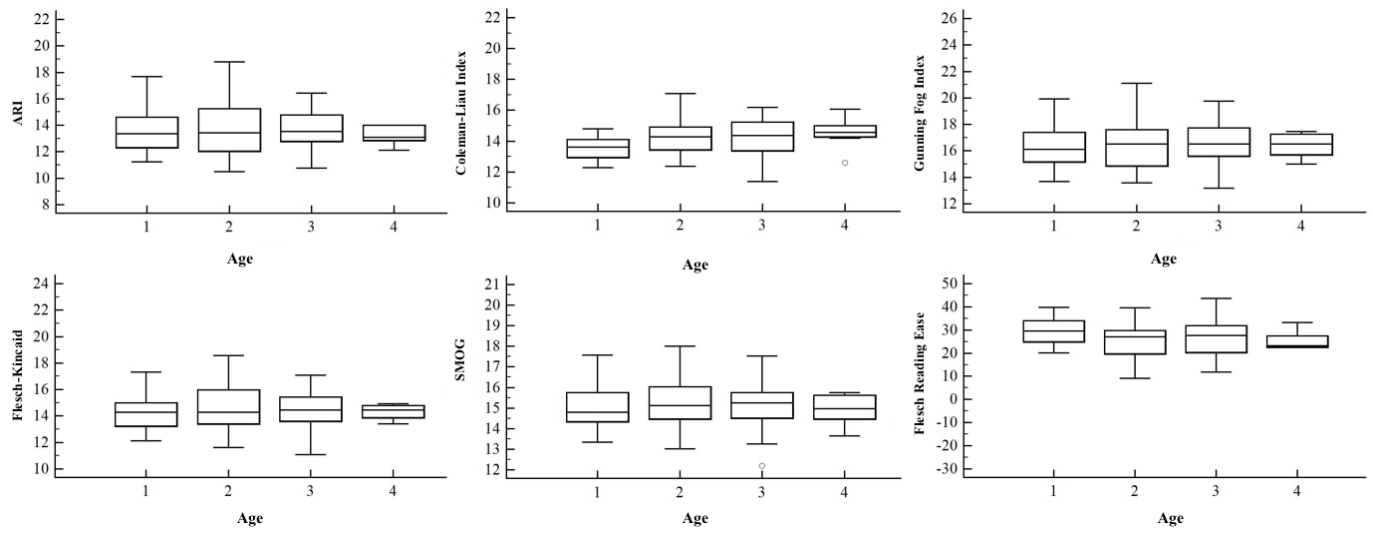
Correlation between age (where 1 is 0-20 years, 2 is 20-40 years, 3 is 40-60 years, and 4 is 60-80 years) at onset of autoimmune diseases and the readability scores of their corresponding Wikipedia pages. Error bars indicate SD. ARI: Automated Readability Index, SMOG: Simple Measure of Gobbledygook.

**Figure 2:**
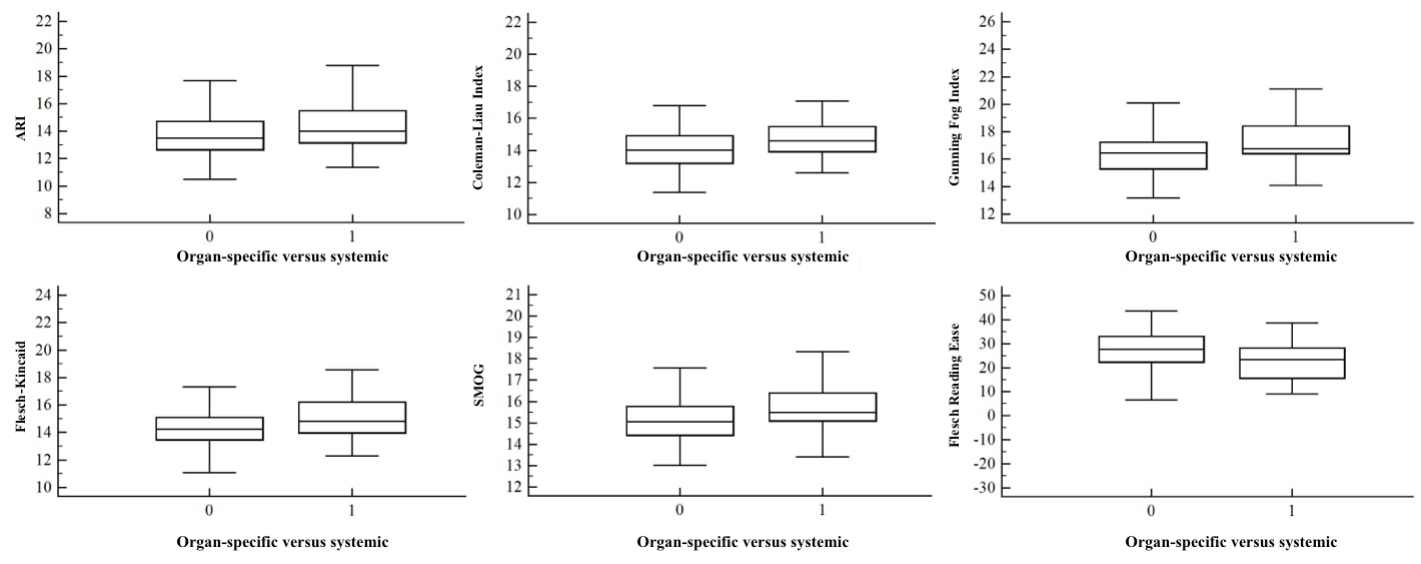
Correlation between pathological characteristics (0=organ-specific, 1=systemic) of autoimmune diseases and the readability scores of their corresponding Wikipedia pages. Error bars indicate SD. ARI: Automated Readability Index, SMOG: Simple Measure of Gobbledygook.

**Figure 3:**
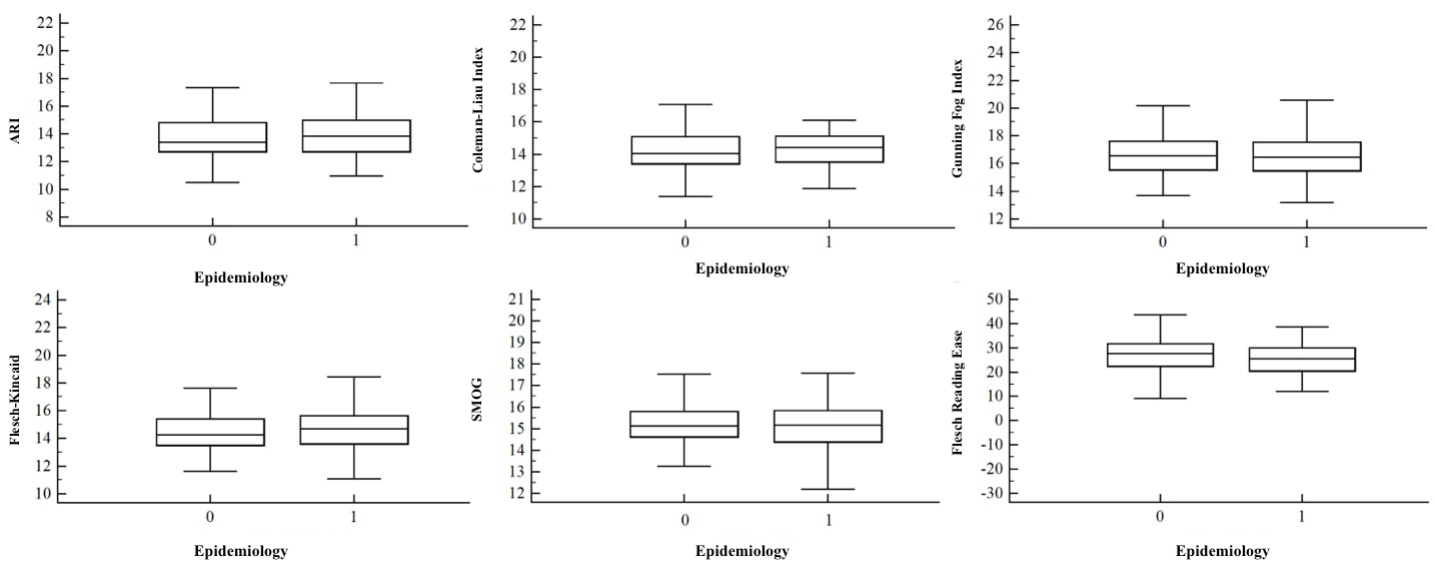
Correlation between the epidemiology of autoimmune diseases (0=not rare, 1=rare) and the readability scores of their corresponding Wikipedia pages. Error bars indicate SD. ARI: Automated Readability Index, SMOG: Simple Measure of Gobbledygook.

**Figure 4:**
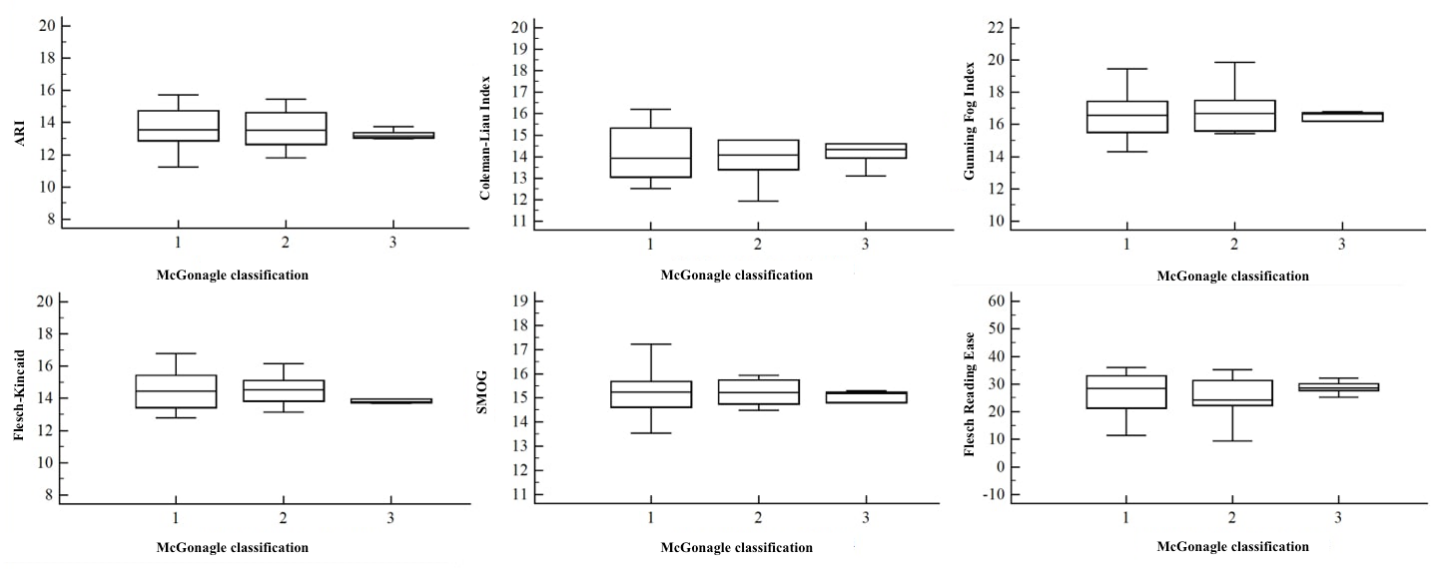
Correlation between McGonagle classification of autoimmune diseases (1=classic polygenic autoimmune disease, 2=polygenic autoinflammatory disease, 3=mixed-pattern disease) and the readability scores of their corresponding Wikipedia pages. Error bars indicate SD. ARI: Automated Readability Index, SMOG: Simple Measure of Gobbledygook.

Both univariate and multivariate analyses (Table 2,Figure 1, Figure 2, Figure 3, and Figure 4) demonstrated no correlation between readability scores and clinical parameters, epidemiological features of autoimmune disorders in terms of etiopathogenesis, incidence (common vs rare), and age of onset (0-20 years vs 20-40 years vs 40-60 years).

**Table 1:** Readability scores of autoimmune disorder-related Wikipedia pages.

Readability index		Mean	SD
**Approximate representation of the US grade level needed to comprehend the text**			
	Automated Readability Index	14.06	2.12
	Coleman-Liau Index	14.48	1.57
	Flesch-Kincaid Grade Level	14.86	1.95
	Simple Measure of Gobbledygook	15.38	1.37
**Number of years of formal education required to easily understand the text on first reading**			
	Gunning Fog Index	16.87	2.03
**Reading Ease Index (score 0-100)**			
	Flesch Reading Ease	24.34	10.73

**Table 2:** Multivariate regression analysis of the readability scores for the autoimmunity disorder-related Wikipedia pages.

Readability index	Parameter	B	SD	*t* statistic	*P* value
**Gunning Fog Index**					
	Intercept	17.032	1.174	14.507	<.001
	Classic polygenic autoimmune disease (McGonagle classification)	0.310	0.781	0.397	.69
	Polygenic autoinflammatory disease (McGonagle classification)	0.225	0.852	0.264	.79
	Organ-specific versus systemic	–0.413	0.554	–0.746	.46
	Common versus rare	–0.508	0.627	–0.810	.42
	Age 0-20 years	–0.858	1.083	–0.792	.43
	Age 20-40 years	0.227	0.943	0.240	.81
	Age 40-60 years	–0.130	0.984	–0.132	.90
**Coleman-Liau Index**					
	Intercept	15.162	0.849	17.852	<.001
	Classic polygenic autoimmune disease (McGonagle classification)	0.655	0.565	1.159	.26
	Polygenic autoinflammatory disease (McGonagle classification)	0.165	0.617	0.267	.79
	Organ-specific versus systemic	–0.858	0.401	–2.140	.04^a^
	Common versus rare	–0.335	0.453	–0.738	.47
	Age 0-20 years	–1.237	0.783	–1.579	.13
	Age 20-40 years	–0.304	0.682	–0.445	.66
	Age 40-60 years	–0.706	0.712	–0.992	.33
**Flesch-Kincaid Grade Level**					
	Intercept	14.389	0.930	15.464	<.001
	Classic polygenic autoimmune disease (McGonagle classification)	0.832	0.619	1.343	.19
	Polygenic autoinflammatory disease (McGonagle classification)	0.729	0.676	1.079	.29
	Organ-specific versus systemic	–0.736	0.439	–1.674	.11
	Common versus rare	–0.395	0.497	–0.795	.43
	Age 0-20 years	–0.192	0.858	–0.224	.83
	Age 20-40 years	0.411	0.747	0.550	.59
	Age 40-60 years	–0.152	0.780	–0.195	.85
**Automated Readability Index**					
	Intercept	13.365	1.058	12.636	<.001
	Classic polygenic autoimmune disease (McGonagle classification)	0.437	0.704	0.621	.54
	Polygenic autoinflammatory disease (McGonagle classification)	0.430	0.768	0.560	.59
	Organ-specific versus systemic	–0.445	0.500	–0.890	.38
	Common versus rare	–0.422	0.565	–0.748	.46
	Age 0-20 years	0.144	0.975	0.147	.88
	Age 20-40 years	0.553	0.849	0.651	.52
	Age 40-60 years	0.127	0.887	0.143	.89
**Simple Measure of Gobbledygook**					
	Intercept	15.095	0.708	21.332	<.001
	Classic polygenic autoimmune disease (McGonagle classification)	0.310	0.471	0.658	.52
	Polygenic autoinflammatory disease (McGonagle classification)	0.303	0.514	0.589	.56
	Organ-specific versus systemic	–0.180	0.334	–0.539	.59
	Common versus rare	–0.170	0.378	–0.450	.66
	Age 0-20 years	–0.264	0.653	–0.404	.70
	Age 20-40 years	0.288	0.568	0.506	.62
	Age 40-60 years	–0.065	0.593	–0.110	.91
**Flesch Reading Ease**					
	Intercept	22.710	5.227	4.344	<.001
	Classic polygenic autoimmune disease (McGonagle classification)	–6.342	3.479	–1.823	.08
	Polygenic autoinflammatory disease (McGonagle classification)	–4.004	3.796	–1.055	.30
	Organ-specific versus systemic	6.268	2.469	2.539	.02^a^
	Common versus rare	2.253	2.791	0.807	.43
	Age 0-20 years	5.976	4.821	1.240	.23
	Age 20-40 years	0.294	4.198	0.070	.95
	Age 40-60 years	3.941	4.383	0.899	.38

^a^Statistically significant.

Table 3 shows readability indices broken down by section, and Multimedia Appendix 1 shows pairwise comparisons, corrected with Bonferroni correction for multiple comparisons. Statistically significant differences in readability indices were found among the different sections of the Wikipedia pages related to autoimmune disorders. In particular, considering the Flesch Reading Ease scores, the most readable sections concerned prognosis, while the least readable parts were the diagnostic paragraph(s). The prognostic sections differed from all the other parts, while the diagnostic sections did not differ from the parts on pathogenesis and management. Treatment sections were significantly different only from prognosis sections, while the sections on pathogenesis were significantly different from the prognosis and sections in the “other” category. Finally, these other sections differed from paragraph(s) concerning pathogenesis, diagnosis, and prognosis.

We noted similar, consistent trends for the other readability indices (Table 3,Multimedia Appendix 1).

**Table 3:** Readability scores of autoimmunity disorder-related Wikipedia pages broken down by section.

Readability index	Section	Mean	SD

**Flesch Reading Ease**			
	Other	32.15	11.18
	Pathogenesis	25.06	15.27
	Treatment	27.93	12.68
	Diagnosis	23.40	17.41
	Prognosis	40.89	12.62
**Gunning Fog Index**			
	Other	15.45	2.41
	Pathogenesis	17.61	2.71
	Treatment	16.49	2.05
	Diagnosis	17.98	4.60
	Prognosis	14.22	2.37
**Flesch-Kincaid Grade Level**			
	Other	13.53	2.30
	Pathogenesis	15.02	2.90
	Treatment	13.92	2.01
	Diagnosis	15.39	4.77
	Prognosis	11.87	2.22
**Coleman-Liau Index**			
	Other	12.69	2.15
	Pathogenesis	13.77	2.39
	Treatment	14.44	2.36
	Diagnosis	13.95	1.91
	Prognosis	10.79	2.82
**Simple Measure of Gobbledygook**			
	Other	12.21	1.97
	Pathogenesis	13.13	2.12
	Treatment	12.14	1.43
	Diagnosis	13.24	3.14
	Prognosis	10.85	1.67
**Automated Readability Index**			
	Other	12.88	6.10
	Pathogenesis	14.40	3.47
	Treatment	13.52	2.16
	Diagnosis	14.85	5.87
	Prognosis	10.17	2.96

## Discussion

### Principal Findings

To the best of our knowledge, this is the first systematic appraisal of the readability of autoimmune diseases-related Wikipedia material. In this investigation, we found that Wikipedia pages related to autoimmune disorders were characterized by a low level of readability, and this readability level was not correlated in any way with the clinical, pathological, and epidemiological characteristics of the disorders. In other words, the epidemiology (onset age, incidence, prevalence) and clinical presentation of the diseases were not reflected in the readability scores.

Our findings of a low readability of Wikipedia pages are in line with the findings of other studies. In the extant literature, Azer et al [14] found that pages related to cardiovascular diseases were characterized by a readability score of 14.3 (SD 1.7) measured with the Flesch-Kincaid Grade Level, consistent with the readability level typical of university students. Candelario and colleagues, assessing medication guide-related Wikipedia pages, found that Wikipedia medication pages were characterized by a Flesch Reading Ease score of 52.93 and a Flesch-Kincaid Grade Level of 10.26, indicating that the Wikipedia pages were more difficult to read than their corresponding product medication guides [15]. Seth and coworkers [16] systematically investigated the readability of lymphedema-related online material and assessed 152 patient articles. They found an overall mean reading level of 12.6, with individual website reading levels ranging from 9.4 (cancer.org) to 16.7 (wikipedia.org). Interestingly, they noticed that online material describing conservative management differed from that reporting a surgical option in a statistically significant way (reading level of 12.7 vs 15.6, respectively, *P*<.001) [16]. Brigo and coworkers [17] computed the readability level of websites concerning epilepsy and obtained a difficult to fairly difficult readability level of 44.0±8.2, as measured with the Flesch Reading Ease instrument, with text readability corresponding to an 11th academic grade level (mean 11.3, SD 1.9). In particular, focusing on the Wikipedia pages, the average Flesch Reading Ease score was 25.6 (SD 9.5), with the other readability scales corresponding to a 14th grade level (mean 14.3, SD 1.7) [17]. Thomas et al [18] assessed the readability of Wikipedia pages related to renal diseases and found that they were written at a university reading level. Similar findings were obtained by other scholars. Tulbert and colleagues [19], in their analysis of dermatological online resources, compared 3 popular websites providing patient-education material (WebMD.com, Wikipedia.org, and MedicineOnline.com) versus the online material produced by the American Academy of Dermatology. Rajagopalan and coworkers [20], in their appraisal of patient-oriented cancer information on the Internet, compared Wikipedia with a professionally maintained database and found that the latter was more readable (Flesch-Kincaid Grade Level 14.1 vs 9.6, respectively). Azer [21] assessed 39 Wikipedia articles related to gastroenterology and hepatology and computed a mean overall readability score of 26 (SD 9.0) (range –8.0, SD 55.7 to 44.4, SD 1.4). Volsky and coauthors [22], in their analysis of pediatric otolaryngology online resources, compared Wikipedia, eMedicine, and MedlinePlus. They found that Wikipedia was the least readable resource. Brigo and Erro [23] analyzed the Wikipedia page related to Parkinson disease and found a low readability level (Flesch Reading Ease score 30.31).

Very recently, a collateral Wikipedia project named Simple English Wikipedia has been developed and implemented with the aim of helping users to better read and understand uncommon, unfamiliar, or complex topics, by simplifying the Wikipedia articles. Pages written in simple English use fewer words and easier grammar than those written in standard English. Although generally aimed at students or children, Simple English Wikipedia may be helpful for people with low literacy skills, even though these pages are less accessed than those written in standard English. Therefore, the initiative of the Simple English Wikipedia should be further encouraged. For example, a direct link to it should be added in each corresponding standard English Wikipedia article, thus supporting and facilitating consultation of Simple English Wikipedia.

Other similar ongoing projects include the WikiProject Epilepsy, specifically focused on epilepsy-related Wikipedia pages. Under the leadership of the International League Against Epilepsy and a committee of well-known scientists including Günter Krämer, Selim Benbadis, and Nicola Maggio, existing epilepsy-related entries are being critically revised and edited, and as well new entries are being created to provide the public with a comprehensive, clearly accessible database including both articles and video sequences of seizure episodes with commentary.

Another project, a collaboration of the Imperial College School of Medicine (ICSM) and the WikiProject Medicine (the so-called ICSM Wikipedia Easter Project), aimed to bring together medical students and professors, librarians, and technologists in a 2-day competition to edit and provide updated content for a selected medical topic. However, this project, like the WikiProject University of California, was intended as a challenge rather than as a long-lasting initiative.

Professionals working in the field of health care, helped by public and specialized libraries, as well as by other stakeholders, should make efforts to design or to bookmark online health-related material that, while preserving a high quality, accuracy, and exhaustiveness, can be easily read and understood [9].

Moreover, a particular attempt should be made to simplify information on pathologies with a low incidence and prevalence and with a complex pathogenesis, also taking into account onset age, since the Internet presents a highly valued opportunity of supporting patients with rare autoimmune diseases [24].

In the field of autoimmune diseases, to the best of our knowledge, no eHealth initiatives exist with the goal of improving the quality of disease-specific online material. Our study has contributed to drawing attention to the low readability content of autoimmune disease-related Wikipedia pages, emphasizing the urgent need to establish some forms of cooperation between doctors and patient groups working together to make easily accessible disease-specific websites and online materials.

### Conclusions

This study addressed a topic currently neglected in the immunologic research field. While other medical fields have addressed the subject of Wikipedia readability (such as the neurological field), there is a dearth of similar studies in the specialties of immunology and autoimmunology. Our investigation was aimed at filling this gap in knowledge. Our findings underline the need for accessible and easily understandable online material dedicated to autoimmune disorders to further improve patients’ health literacy and awareness of their conditions. Even though readability metrics are only an indirect measure of literacy skills, being reliable and validated, they can provide a first objective assessment of the complexity of written material, guiding the process of customizing online health-related material to the patient’s reading level and, thus, enhancing patient-centered communication [25,26].

## Appendix

Multimedia Appendix 1 One-way analysis of variance.

## Abbreviations

AARDA: American Autoimmune Related Diseases Association
ARI: Automated Readability Index
ICSM: Imperial College School of Medicine
SMOG: Simple Measure of Gobbledygook
