# Supplementary material for: Readability of Wikipedia Pages on Autoimmune Disorders: Systematic Quantitative Assessment
Source: J Med Internet Res. 2017 Jul 18;19(7):e260. doi: 10.2196/jmir.8225 (PMC5539385; doi:10.2196/jmir.8225)
Supplement: Multimedia Appendix 1 [file jmir_v19i7e260_app1.pdf]

Results of 1-way analysis of variance for each readability score of the autoimmunity disorder-related Wikipedia pages broken down by section.

Readability index	Section	Pairwise comparison	Mean difference	*P* value
**Flesch Reading Ease**				
	Other	Pathogenesis	7.10[a]	.004
		Treatment	4.23	.29
		Diagnosis	8.75[a]	<.001
		Prognosis	–8.73[a]	.001
	Pathogenesis	Other	–7.10[a]	.004
		Treatment	–2.87	>.99
		Diagnosis	1.65	>.99
		Prognosis	–15.83[a]	<.001
	Treatment	Other	–4.23	.29
		Pathogenesis	2.87	>.99
		Diagnosis	4.52	.21
		Prognosis	–12.96[a]	<.001
	Diagnosis	Other	–8.75[a]	<.001
		Pathogenesis	–1.65	>.99
		Treatment	–4.52	.21
		Prognosis	–17.48[a]	<.001
	Prognosis	Other	8.73[a]	.001
		Pathogenesis	15.83[a]	<.001
		Treatment	12.96[a]	<.001
		Diagnosis	17.48[a]	<.001
**Gunning Fog Index**				
	Other	Pathogenesis	–2.15[a]	<.001
		Treatment	–1.03	.14
		Diagnosis	–2.52[a]	<.001
		Prognosis	1.24	.10
	Pathogenesis	Other	2.15[a]	<.001
		Treatment	1.12	.13
		Diagnosis	–0.37	>.99
		Prognosis	3.39[a]	<.001
	Treatment	Other	1.03	.14
		Pathogenesis	–1.12	.13
		Diagnosis	–1.49[a]	.005
		Prognosis	2.27[a]	<.001
	Diagnosis	Other	2.52[a]	<.001
		Pathogenesis	0.37	>.99
		Treatment	1.49[a]	.005
		Prognosis	3.76[a]	<.001
	Prognosis	Other	–1.24	.10
		Pathogenesis	–3.39[a]	<.001

		Treatment	–2.27[a]	<.001
		Diagnosis	–3.76[a]	<.001
**Flesch-Kincaid Grade Level**				
	Other	Pathogenesis	–1.49[a]	.007
		Treatment	–0.40	>.99
		Diagnosis	–1.87[a]	<.001
		Prognosis	1.66[a]	.007
	Pathogenesis	Other	1.49[a]	.007
		Treatment	1.09	.17
		Diagnosis	–0.38	>.99
		Prognosis	3.15[a]	<.001
	Treatment	Other	0.40	>.99
		Pathogenesis	–1.09	.17
		Diagnosis	–1.47[a]	.007
		Prognosis	2.06[a]	.001
	Diagnosis	Other	1.87[a]	<.001
		Pathogenesis	0.38	>.99
		Treatment	1.47[a]	.007
		Prognosis	3.53[a]	<.001
	Prognosis	Other	–1.66[a]	.007
		Pathogenesis	–3.15[a]	<.001
		Treatment	–2.06[a]	.001
		Diagnosis	–3.53[a]	<.001
**Coleman-Liau Index**				
	Other	Pathogenesis	–1.08[a]	.009
		Treatment	–1.75[a]	<.001
		Diagnosis	–1.25[a]	<.001
		Prognosis	1.90[a]	<.001
	Pathogenesis	Other	1.08[a]	.009
		Treatment	–0.67	.47
		Diagnosis	–0.17	>.99
		Prognosis	2.98[a]	<.001
	Treatment	Other	1.75[a]	<.001
		Pathogenesis	0.67	.47
		Diagnosis	0.50	>.99
		Prognosis	3.65[a]	<.001
	Diagnosis	Other	1.25[a]	<.001
		Pathogenesis	0.17	>.99
		Treatment	–0.50	>.99
		Prognosis	3.16[a]	<.001
	Prognosis	Other	–1.90[a]	<.001
		Pathogenesis	–2.98[a]	<.001
		Treatment	–3.65[a]	<.001
		Diagnosis	–3.16[a]	<.001
**Simple Measure of Gobbledygook**				
	Other	Pathogenesis	–0.92[a]	.03

		Treatment	0.07	>.99
		Diagnosis	–1.03[a]	.005
		Prognosis	1.36[a]	.001
	Pathogenesis	Other	0.92[a]	.03
		Treatment	0.99[a]	.03
		Diagnosis	–0.11	>.99
		Prognosis	2.28[a]	<.001
	Treatment	Other	–0.07	>.99
		Pathogenesis	–0.99[a]	.03
		Diagnosis	–1.10[a]	.004
		Prognosis	1.29[a]	.004
	Diagnosis	Other	1.03[a]	.005
		Pathogenesis	0.11	>.99
		Treatment	1.10[a]	.004
		Prognosis	2.39[a]	<.001
	Prognosis	Other	–1.36[a]	.001
		Pathogenesis	–2.28[a]	<.001
		Treatment	–1.29[a]	.004
		Diagnosis	–2.39[a]	<.001
**Automated Readability Index**				
	Other	Pathogenesis	–1.52	.21
		Treatment	–0.64	>.99
		Diagnosis	–1.97[a]	.01
		Prognosis	2.71[a]	.002
	Pathogenesis	Other	1.52	.21
		Treatment	0.88	>.99
		Diagnosis	–0.45	>.99
		Prognosis	4.23[a]	<.001
	Treatment	Other	0.64	>.99
		Pathogenesis	–0.88	>.99
		Diagnosis	–1.32	.41
		Prognosis	3.36[a]	<.001
	Diagnosis	Other	1.97[a]	.01
		Pathogenesis	0.45	>.99
		Treatment	1.32	.41
		Prognosis	4.68[a]	<.001
	Prognosis	Other	–2.71[a]	.002
		Pathogenesis	–4.23[a]	<.001
		Treatment	–3.36[a]	<.001
		Diagnosis	–4.68[a]	<.001

[a]Statistically significant.
